# Attitudes towards Sharing Digital Footprint Data: a Discrete Choice Experiment

**DOI:** 10.23889/ijpds.v8i3.2287

**Published:** 2023-09-11

**Authors:** Rebecca McDonald, Anya Skatova, Carsten Maple

**Affiliations:** 1 Department of Economics, University of Birmingham; 2 Bristol Medical School, University of Bristol; 3 Warwick Manufacturing Group, Warwick University

## Introduction & Background

Digital footprints data are key for the economy, underpinning business models and service provision. This information can also bring benefit to public good, yet sharing of digital footprints data are predicated on individual attitudes which in term depend on the value these data have to consumers. In this study, we investigated how individuals make decisions about sharing their digital footprints data, as well as which features of the data sharing scenario affect their decision to share the data.

## Objectives & Approach

We used responses from a nationally representative sample of 2,087 UK residents to estimate public preferences towards sharing different types of digital footprint data in scenarios with different features. The main part of our experiment consisted of a Discrete Choice Experiment which allows the relative importance of the different features of data sharing scenarios to be established, revealing the tradeoffs participants make between them. Participants made a series of choices between two hypothetical data sharing scenario options or could “opt out” by choosing neither specified option. For example, we examined the differences in responses when data are shared for different purposes (e.g., for research vs private benefit), as well as when data are shared with more or less granular details about identity or location. The data were analysed using a logistic regression with an alternative-specific constant.

## Relevance to Digital Footprints

We focused on understanding whether varied features of six different types of digital footprints data - namely banking transactions, electricity use at home, retail loyalty cards use, browsing history, social media, and physical activity data - affect people’s decision whether to share these data.

## Results

Participants were more likely to share their data with a university for academic research than with a private company or government. Participants were also most reluctant to share data alongside their personal identity. Participants were concerned with the recipient of the data and their purpose in requesting it; whether the data would be shared along with their location and if so, to what specificity; and with the level of aggregation of the data (i.e. whether it would be shared in fine detail or as a monthly summary). In addition, we demonstrated the importance of the type of data to be shared, with people most reluctant to share bank transactions data, but relatively unconcerned about sharing their physical activity, electricity use and loyalty cards data.

## Conclusions & Implications

We contribute by highlighting the trade-offs individuals are willing to make between different elements of a data sharing situation, and the relative importance of these different aspects. We also demonstrate that individuals’ have positive attitudes to share digital footprints data for research benefiting public good. By integrating these preferences into ethical and responsible research models, we can create fairer and more balanced data sharing frameworks, which can ultimately help people to make better choices about their personal digital footprints data.

